# Targeting of histone methyltransferase DOT1L plays a dual role in chemosensitization of retinoblastoma cells and enhances the efficacy of chemotherapy

**DOI:** 10.1038/s41419-021-04431-y

**Published:** 2021-12-09

**Authors:** Yu Mao, Yu Sun, Zhixuan Wu, Jingzhi Zheng, Jianing Zhang, Jiaqi Zeng, Chunsik Lee, Jong Kyong Kim

**Affiliations:** State Key Laboratory of Ophthalmology, Zhongshan Ophthalmic Center, Sun Yat-sen University, 510060 Guangzhou, China

**Keywords:** Chemotherapy, Eye cancer, Oncogenes, Paediatric cancer

## Abstract

Aberrant and exclusive expression of chromatin regulators in retinoblastoma (RB) in contrast to terminally differentiated normal retina presents a unique opportunity of selective targeting for RB. However, precise roles of these chromatin regulators in RB development and their potential as therapeutic targets have not been defined thoroughly. Here, we report that targeting of disruptor of telomeric silencing 1-like (DOT1L), a histone H3K79 methyltransferase, sensitizes RB cells to chemotherapeutic drugs by impairing the DNA damage response and thereby potentiating apoptosis while it is largely inefficacious as a single-agent therapy. Moreover, we identified high mobility group AT-hook 2 (HMGA2) as a novel DOT1L target gene in RB cells and found that its aberrant expression is dependent on DOT1L. As HMGA2 depletion reduced CHK1 phosphorylation during DNA damage response and augmented the drug sensitivity in RB cells, our results suggested that DOT1L targeting has a dual role in chemosensitization of RB cells by directly interfering with the immediate involvement of DOT1L in early DNA damage response upon genotoxic insults and also by downregulating the expression of HMGA2 as a rather late effect of DOT1L inhibition. Furthermore, we provide the first preclinical evidence demonstrating that combined therapy with a DOT1L inhibitor significantly improves the therapeutic efficacy of etoposide in murine orthotopic xenografts of RB by rendering the response to etoposide more potent and stable. Taken together, these results support the therapeutic benefits of DOT1L targeting in combination with other chemotherapeutic agents in RB, with mechanistic insights into how DOT1L targeting can improve the current chemotherapy in an RB cell-selective manner.

## Introduction

Retinoblastoma (RB) is a developmental tumor occurring in the eyes of young children through multiple genetic and epigenetic alterations following the initiating lesions in the *RB1* gene in the developing retina [1–3]. Consistent with the implication of epigenetic dysregulation in RB development, a plethora of chromatin regulators have been identified to be misregulated in human RB whereas these aberrantly expressed chromatin regulators are not expressed in normal retina [4].

Chemotherapy is an important therapeutic modality for RB treatment and most chemotherapeutic regimens include conventional genotoxic or cytotoxic drugs due to the lack of targeted drugs for RB [5]. Considering the possible adverse effects of conventional chemotherapeutic drugs [6–8], strategies to selectively sensitize RB cells to these drugs have been searched as an alternative approach to improve the efficacy of current chemotherapy. In line with this rationale, we recently demonstrated that downmodulation of UHRF1, an epigenetic regulator, enhances the sensitivity to chemotherapeutic drugs in RB cells by altering distinct sets of effector genes involved in DNA repair and redox homeostasis [9, 10]. Among the chromatin regulators misregulated in RB, this study focuses on histone methyltransferase DOT1L to explore its role in RB cell chemosensitization.

DOT1L is the only known histone methyltransferse catalyzing H3K79 methylation which is an activating mark for gene transcription by promoting transcriptional elongation [11, 12]. From the perspective of cancer research, DOT1L has been perceived as a promising therapeutic target for mixed lineage leukemia (MLL)-rearranged leukemia since DOT1L was found to be recruited by various MLL-fusion proteins to MLL target genes such as *HOXA9* and *MEIS1*, resulting in aberrant H3K79 methylation and consequential expression of these leukemogenic genes [13–15]. In addition to leukemia, DOT1L has been reported as a potential therapeutic target in other solid tumors including breast and prostate cancer [16, 17]. Furthermore, DOT1L and H3K79 methylation were found to be essential for ionizing radiation (IR)-induced 53BP1 foci formation during G1/G2 phase, and DOT1L depletion in cancer cells led to prolonged presence of phosphorylated histone H2AX on Ser139 (γH2AX) post-IR, suggestive of defective DNA repair [18]. Consistently, pharmacological inhibition of DOT1L in combination with DNA-damaging agents further enhanced the growth inhibition of colorectal cancer cells and MLL-rearranged leukemia cells [19, 20].

The functions of misregulated chromatin regulators in RB have just begun to be understood in recent years [4]. However, the role of HMGA2 in RB cell proliferation was noted nearly two decades ago, along with the observation that HMGA2 expression is high in murine embryonic retina and human RB tumors but is not detectable in terminally differentiated retina [21]. HMGA2 is a chromatin-associated protein which can modulate transcription by altering the chromatin structure and affinity of transcription factors, and ectopic expression of HMGA2 is known to induce neoplastic transformation and tumorigenesis [22]. Notably, HMGA2 is also involved in the modulation of DNA damage response by altering the expression or phosphorylation of DNA damage checkpoint proteins including ATM and ATR/CHK1 axis, leading to the increased sensitivity to diverse genotoxic insults upon HMGA2 downmodulation in cancer cells [23–25].

In this study, we report that HMGA2 is a novel DOT1L target gene and its aberrant expression in RB cells is dependent on DOT1L, laying a basis for the dual role of DOT1L targeting in chemosensitization of RB cells. Furthermore, we demonstrate that combined therapy with a DOT1L inhibitor significantly improves the efficacy of etoposide in orthotopic xenografts of RB.

## Results

### DOT1L is aberrantly expressed in human RB and pharmacological inhibition of DOT1L sensitizes RB cells to chemotherapeutic drugs

**Fig. 1:**
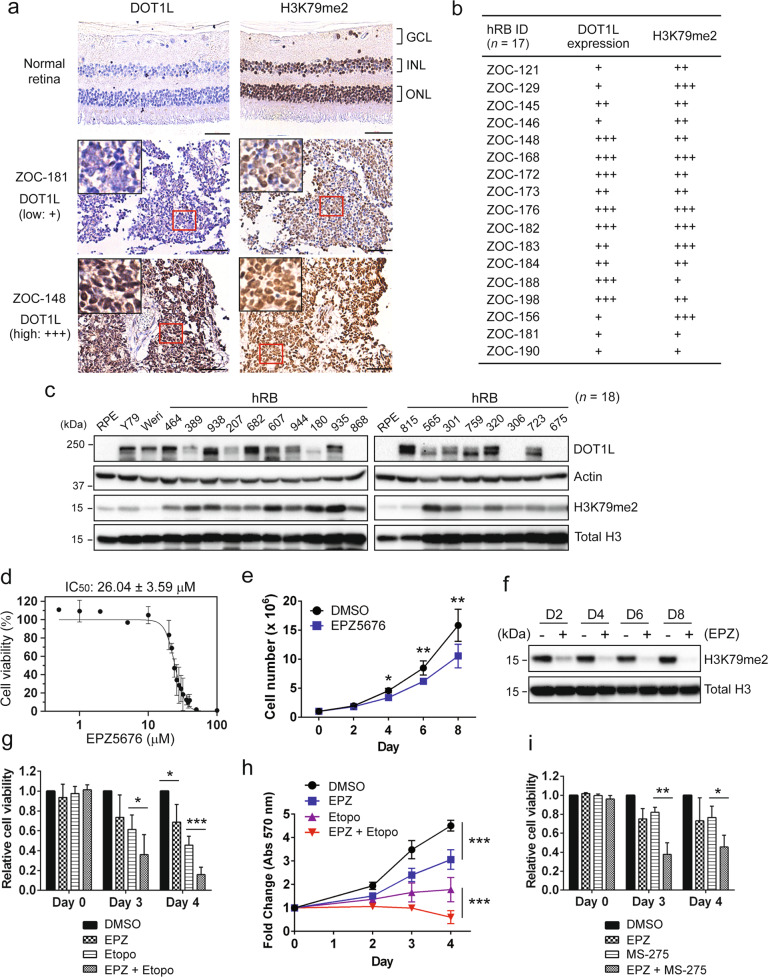
DOT1L is aberrantly expressed in human RB and pharmacological inhibition of DOT1L sensitizes RB cells to chemotherapeutic drugs. **a** Immunostaining of DOT1L and H3K79me2 in serial sections of normal retina and human RB (*n* = 17). The insets are magnified images of the marked regions. GCL ganglion cell layer; INL inner nuclear layer; ONL outer nuclear layer; Scale bar: 50 μm. **b** Summary of DOT1L and H3K79me2 expression by the IHC analysis in **a**. **c** Expression of DOT1L and H3K79me2 in human RB tissue lysates (*n* = 18) and the indicated cell lines. RPE retinal pigment epithelium. **d** Dose-response study for the IC_50_ of EPZ5676 in Y79 cells treated for 48 h by MTT assays. The IC_50_ is shown as the mean ± standard deviation (SD) (*N* = 5). **e** Proliferation of Y79 cells determined by live cell counting in response to 10 µM EPZ5676 for the indicated days in parallel with vehicle control (DMSO). The results represent the mean ± SD (*N* = 3). *P < 0.05; **P < 0.01. **f** Immunoblots for H3K79me2 and total histone H3 in Y79 cells after exposure to 10 µM EPZ5676 for the indicated time. **g**–**i** MTT assays for relative cell viability (**g**, **i**) and proliferation (**h**). Y79 cells were treated with 20 µM EPZ5676 as a single agent or in combination with either 0.5 µM etoposide (**g**, **h**) or 0.5 µM MS-275 (**i**) for the indicated time. The data are shown as the mean ± SD (*N* = 3). *P < 0.05; **P < 0.01; ***P < 0.001.

We examined whether DOT1L is highly expressed in our larger cohorts of human RB as reported for a small number of RB samples [26]. While there was no detectable expression of DOT1L in normal retina, the majority of human RB showed aberrant expression of DOT1L at varying levels, along with Y79 and Weri-Rb1 RB cell lines (Fig. 1a–c and Fig. S1). The DOT1L expression showed a moderate correlation with H3K79 methylation in the tumor sections (Fig. 1a, b and Fig. S1). Of note, H3K79 methylation was detected in normal retina in the absence of DOT1L expression, indicating that H3K79 methylation marks deposited during retinal development persist in the terminally differentiated retina (Fig. 1a). In tumor tissue lysates, there was no clear correlation between the DOT1L expression and H3K79 methylation, suggesting that aberrant expression of DOT1L in RB tumors does not exert much effect on global H3K79 methylation (Fig. 1c). We then examined whether inhibition of DOT1L would have any growth-inhibitory effects on RB cells by determining the IC_50_ of EPZ5676, a small-molecule inhibitor of DOT1L, in Y79 cells (Fig. 1d). When Y79 cells were treated with EPZ5676 up to 8 days, cells started showing a significant decrease in numbers from day 4 (Fig. 1e). EPZ5676 treatment was highly efficient to reduce H3K79 methylation without causing any significant changes in the DOT1L protein level (Fig. 1f and Fig. S2). These results demonstrated that EPZ5676 treatment alone can decrease proliferation of Y79 cells over time, but the significant growth-inhibitory effects of EPZ5676 seemed to require either a higher dose or longer treatment time than other chemotherapeutic drugs such as etoposide and histone deacetylase (HDAC) inhibitors [10]. As DOT1L is known to be required for proper DNA damage response and repair [18, 19, 27], we examined if inhibition of DOT1L can sensitize RB cells to genotoxic drugs. As expected, cotreatment of etoposide with EPZ5676 further reduced RB cell viability and proliferation (Fig. 1g, h). Similarly, DOT1L inhibition was found to increase the sensitivity to an HDAC inhibitor MS-275 in RB cells (Fig. 1i).

### Enhanced drug sensitivity upon DOT1L inhibition involves augmented apoptosis

**Fig. 2:**
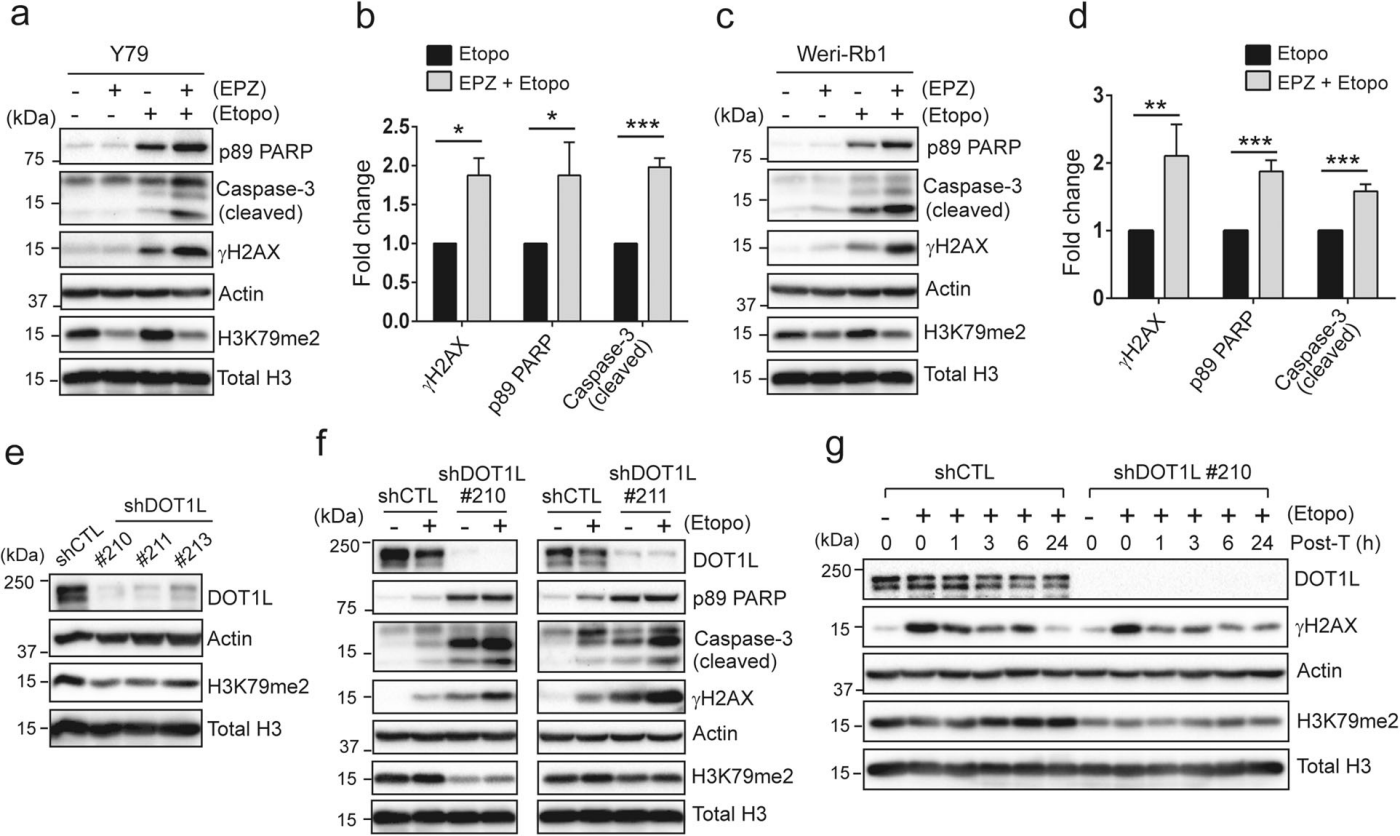
Enhanced drug sensitivity upon DOT1L inhibition involves augmented apoptosis. **a**, **c** Immunoblots for indicated proteins in Y79 cells subjected to single or combined treatment of 20 µM EPZ5676 and 0.5 µM etoposide for 48 h (**a**) and in Weri-Rb1 cells treated for 20 h (**c**). **b**, **d** Densitometric analyses of the indicated proteins in **a** and **c**, respectively. The graph is shown as the mean ± SD of fold changes of the cotreatment group, relative to the normalized level in etoposide-treated cells (*N* = 3). *P < 0.05; **P < 0.01; ***P < 0.001. **e** Lentiviral knockdown of DOT1L in Y79 cells. **f** Immunoblots in Y79 shDOT1L cells treated with 0.5 µM etoposide for 48 h. **g** Immunoblots showing the recovery kinetics from acute DNA damage. Y79 control and shDOT1L cells were treated with 10 μM etoposide for 1 h, and then placed in fresh media without the drug for the indicated time post-treatment (post-T) to allow the recovery.

We next determined if the increased sensitivity to etoposide upon DOT1L inhibition accompanies higher apoptosis by examining the cleaved caspase-3 and poly (ADP-ribose) polymerase (PARP). While single EPZ5676 treatment did not cause any significant apoptosis and DNA damage, cotreatment with etoposide generated higher apoptosis and double-strand breaks (DSBs) than single etoposide treatment (Fig. 2a, b). Similar enhancement in apoptosis and DNA damage was observed for Weri-Rb1 cells (Fig. 2c, d). Notably, etoposide treatment caused a modest increase in total H3K79 methylation levels, verifying that DOT1L activity is indeed implicated in the DNA damage response (Fig. S3). We then depleted DOT1L in Y79 cells by lentiviral gene knockdown to examine whether protein depletion has the similar effects on the drug sensitivity. Both of the tested DOT1L-knockdown cells showed an increased sensitivity to etoposide in comparison with control-knockdown cells (Fig. 2e, f). Furthermore, DOT1L-depleted cells were found to be inefficient to clear the γH2AX compared to control cells which resolved the DNA damage signal to the background level at 24 h of the recovery time after acute DNA damage (Fig. 2g). Taken together, these data demonstrate that DOT1L inhibition or depletion in RB cells increases the sensitivity to DNA-damaging agents by impairing the DNA damage response and thereby enhancing apoptosis.

### HMGA2 expression is downregulated by DOT1L inhibition

**Fig. 3:**
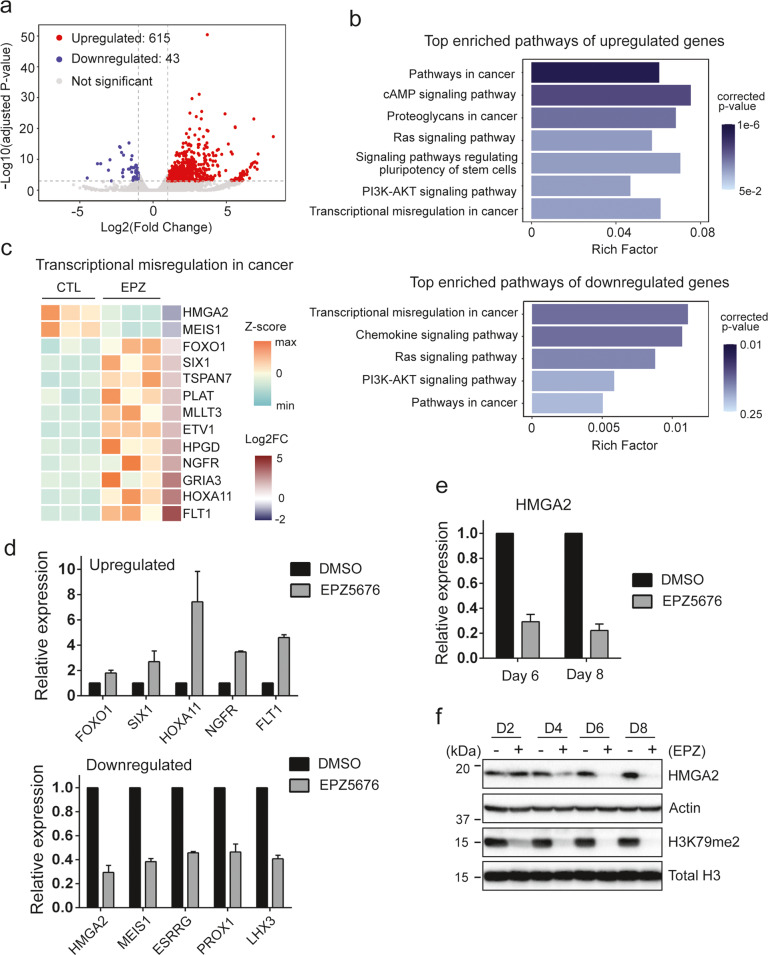
HMGA2 expression is downregulated by DOT1L inhibition. **a** Volcano plot of DEGs in EPZ5676-treated Y79 cells in comparison to vehicle-treated cells. **b** KEGG pathway enrichment analyses for upregulated and downregulated DEGs. Cancer-related pathways are shown from the top 20 enriched pathways ranked by *p*-values before correction. The Rich Factor indicates the gene count ratio of DEG/annotated genes belonging to each pathway term listed. **c** Heat map of DEGs belonging to the KEGG pathway term of transcriptional misregulation in cancer. Each column represents an independent replicate. **d** qRT-PCR analysis for the relative expression of indicated DEGs in Y79 cells treated with 10 µM EPZ5676 for 6 days. The bar graph is shown as the mean ± SD of fold changes, relative to the vehicle-treated group (*N* = 3). **e**, **f** Expression of HMGA2 determined by qRT-PCR (**e**) and immunoblot (**f**) analyses for Y79 cells treated with 10 µM EPZ5676 for the indicated days. The bar graph represents the mean ± SD of fold changes (*N* = 4).

The growth-inhibitory effect of EPZ5676 as a single agent was detectable only at much later time points in RB cells while DOT1L inhibition based on the H3K79 methylation status was highly effective as early as day 2 of the treatment (Fig. 1e, f). This observation led us to a possibility that the later effects of EPZ5676 on cell proliferation may be driven by epigenetic expression changes of certain genes targeted by DOT1L. To identify the genes that are transcriptionally regulated by EPZ5676 treatment, we performed RNA-sequencing on Y79 cells treated with EPZ5676 for 6 days in comparison with vehicle-treated cells, which allowed us to identify total 658 differentially expressed genes (DEGs) (Fig. 3a and Table S1). Our pathway analysis on the DEGs revealed that many cancer-related pathways are significantly enriched (Fig. 3b), and several DEGs were shown to participate in transcriptional misregulation in cancer (Fig. 3c). This result was further supported by the Gene Ontology analysis (Figs. S4 and S5). A subset of the DEGs involved in transcriptional regulation was further validated by independent gene expression analyses (Fig. 3d and Table S2). Among these DEGs, we focused on the downregulated genes to identify the potential DOT1L target genes whose expression would be directly and also negatively regulated by EPZ5676 treatment. In this regard, *HMGA2* was considered as the top candidate gene for a further investigation since inhibition of HMGA2 expression has been shown to reduce RB cell proliferation [21, 28]. Moreover, HMGA2 is known to be involved in the DNA damage response and modulation of chemosensitivity in cancer cells in addition to its role in transcriptional regulation as a chromatin-associated protein [22–25]. When we monitored the expression changes of HMGA2 by EPZ5676 treatment, there was a gradual decrease at both transcript and protein levels over time (Fig. 3e, f), suggesting that *HMGA2* gene may get epigenetically repressed by DOT1L inhibition. Furthermore, the significant reduction of HMGA2 protein from day 4 correlated with the proliferation-inhibitory effect of EPZ5676 observed from day 4 (Figs. 1e and 3f).

### HMGA2 is a target gene of DOT1L

**Fig. 4:**
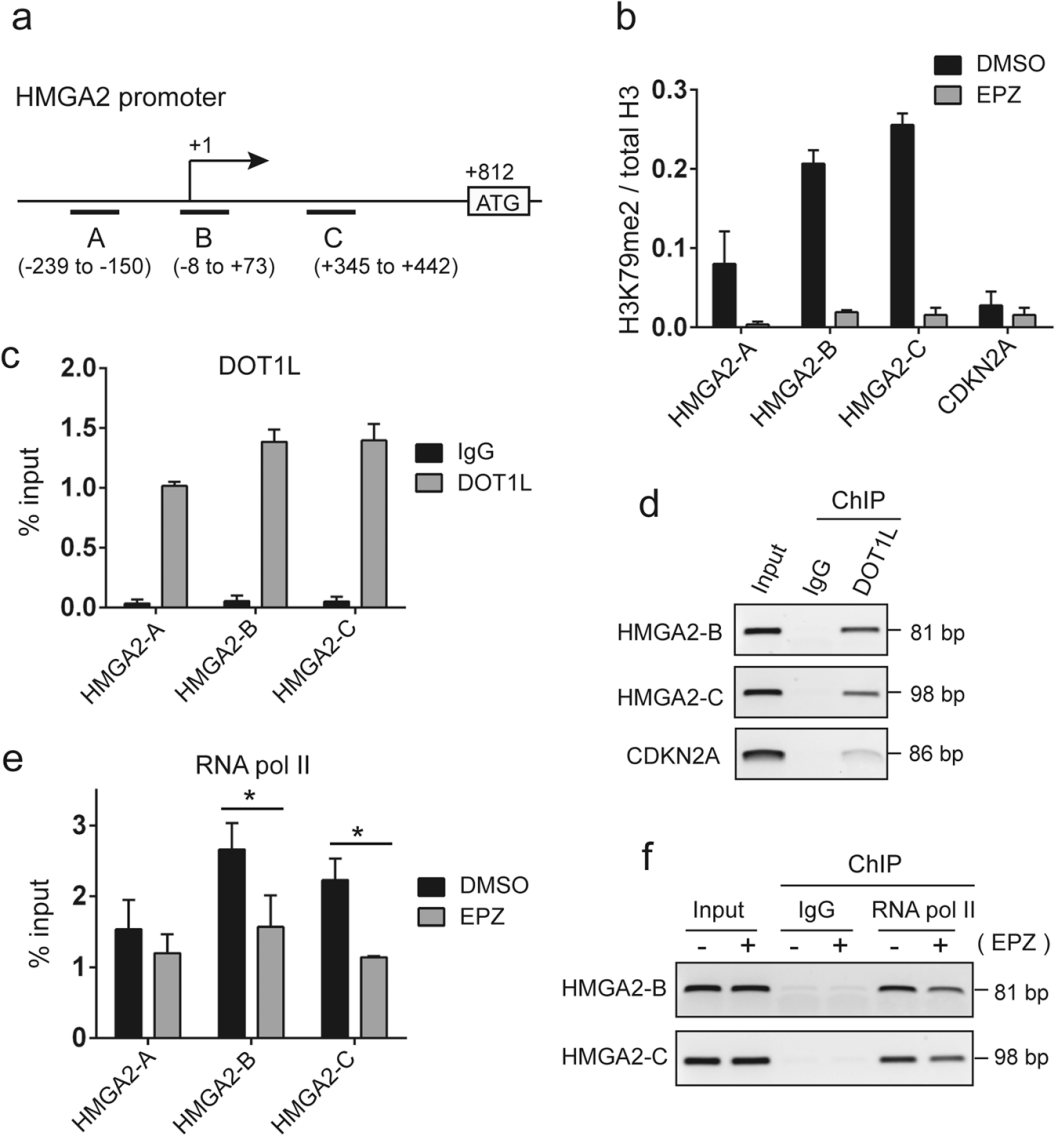
HMGA2 is a DOT1L target gene. **a** Schematic of the *HMGA2* promoter structure near the transcription start site, with the location of primers used for ChIP assays. **b** ChIP-qPCR analysis for H3K79me2 enrichment at different locations of the *HMGA2* promoter. Y79 cells were treated with 10 µM EPZ5676 for 6 days before the ChIP assays. The *CDKN2A* promoter is repressed in Y79 cells and shown as a negative control. The data represent the mean ± SD of normalized H3K79me2/total H3 ratios (*N* = 3). **c**, **d** DOT1L binding at the *HMGA2* promoter determined by ChIP-qPCR (**c**) and PCR-gel (**d**) analyses. The graph is shown as the mean ± SD of % input (*N* = 3). **e**, **f** RNA pol II association at the *HMGA2* promoter determined by ChIP-qPCR (**e**) and PCR-gel (**f**) analyses in Y79 cells treated with 10 µM EPZ5676 for 6 days. The graph represents the mean ± SD of % input (*N* = 3). *P < 0.05.

The gradual decrease in HMGA2 expression by EPZ5676 treatment suggested that *HMGA2* gene may be regulated by the H3K79 methylation status at the promoter as histone H3K79 methylation is generally associated with transcriptional upregulation [12]. Chromatin immunoprecipitation assays revealed that *HMGA2* promoter was indeed decorated with H3K79 methylation marks in vehicle-treated Y79 cells, which were dramatically reduced by EPZ5676 treatment in contrast to the case of *CDKN2A* shown as a repressed gene promoter in RB cells (Fig. 4a, b). We could also confirm that DOT1L binds to the *HMGA2* promoter regions (Fig. 4c, d). In agreement with a previous report on other DOT1L target genes [29], H3K79 methylation marks and DOT1L binding turned out to be highly enriched at the transcription start site (primer B) and 5′ untranslated region (primer C) of the *HMGA2* promoter (Fig. 4b, c). Interestingly, RNA polymerase II (pol II) association was enriched at the same regions of the promoter where high H3K79 methylation was detected, and EPZ5676 treatment significantly reduced the association of RNA pol II at the regions (Fig. 4e, f). These results demonstrate that HMGA2 is a DOT1L target gene and its expression is epigenetically regulated by DOT1L in RB cells.

### HMGA2 downregulation contributes to the anticancer effects of DOT1L inhibition

**Fig. 5:**
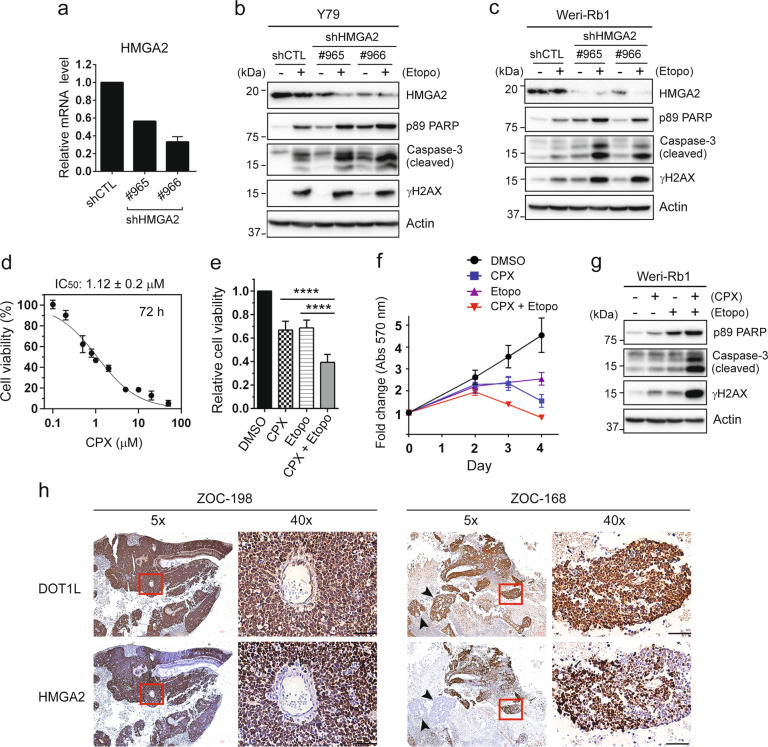
HMGA2 downregulation contributes to the anticancer effects of DOT1L inhibition. **a** Lentiviral HMGA2 knockdown in Y79 cells determined by qRT-PCR (*N* = 2). **b**, **c** Immunoblots for the indicated proteins in Y79 shHMGA2 cells treated with 10 µM etoposide for 24 h (**b**) and in Weri-Rb1 shHMGA2 cells treated with 0.5 µM etoposide for 20 h (**c**). **d** Dose-response study for the IC_50_ of CPX in Y79 cells treated for 72 h by MTT assays. The IC_50_ is shown as the mean ± SD (*N* = 6). **e**, **f** MTT assays for relative cell viability and proliferation. Y79 cells were subjected to single or combined treatment of 0.5 µM CPX and 0.2 µM etoposide for 72 h to determine the relative cell viability (**e**) or up to 4 days to monitor the cell proliferation (**f**). The results are shown as the mean ± SD (*N* = 6). ****P < 0.0001. **g** Immunoblots for indicated proteins in Weri-Rb1 cells treated with 2 µM CPX and 0.5 µM etoposide for 20 h. **h** Immunostaining of DOT1L and HMGA2 in human RB. Serial sections of the indicated tumor tissues were immunostained for DOT1L and HMGA2. The same region of the serial sections (marked by a red square) was magnified on the right (×40). Arrowheads in ZOC-168 mark DOT1L^+^ HMGA2^−^ tumor foci. Scale bar: 50 μm.

We next investigated whether HMGA2 downmodulation would affect the chemosensitivity of RB cells. Consistent with a previous report [25], HMGA2 knockdown in RB cells increased the sensitivity to etoposide (Fig. 5a–c). As HMGA2 has been known to modulate DNA damage checkpoint proteins in cancer cells [23, 24], we examined the expression and phosphorylation of ATM-CHK2 and ATR-CHK1 upon acute DNA damage in HMGA2-knockdown RB cells (Fig. S6). When cells were allowed to recover from the acute DNA damage induced by etoposide, HMGA2-depleted cells showed defective clearance of γH2AX compared to control cells which removed the DNA damage signal almost completely at 24 h of the recovery time (Fig. S6). While there were no significant changes for the expression and phosphorylation of the ATM-CHK2 axis, we observed that CHK1 phosphorylation was significantly reduced in the HMGA2-depleted cells, suggesting that the impaired CHK1 phosphorylation may account for the inefficient DNA damage response and γH2AX clearance upon HMGA2 downmodulation in RB cells (Fig. S6). The DOT1L-mediated HMGA2 expression in RB cells appeared to play a secondary role in the control of the DNA damage response and drug sensitivity as overexpression of HMGA2 in EPZ5676-treated cells did not attenuate the etoposide-induced apoptosis (Fig. S7). Although HMGA2-specific small-molecule inhibitors are not currently available, a recent drug repurposing approach has identified an antifungal agent ciclopirox (CPX) as a potential inhibitor of HMGA2 [30]. RB cells were sensitive to single CPX treatment (Fig. 5d), and cotreatment with CPX and etoposide generated a greater reduction in cell viability and proliferation along with potentiated apoptosis (Fig. 5e–g). We then checked the expression of DOT1L and HMGA2 on serial sections of human RB. Interestingly, not all DOT1L^+^ regions expressed HMGA2, but HMGA2^+^ regions appeared to be all positive for DOT1L expression (Figs. 5h and S8). These results suggest that DOT1L may be one of the factors regulating the expression of HMGA2 in RB in addition to microRNAs [31, 32], all of which may contribute to the variations in HMGA2 expression in primary tumors. Taken together, these results support our findings that HMGA2 expression is regulated by DOT1L in RB and the HMGA2 downregulation by DOT1L inhibition may constitute another axis of the anticancer activity of DOT1L targeting.

### Cotreatment with a DOT1L inhibitor enhances the therapeutic efficacy of etoposide in orthotopic xenografts of RB

**Fig. 6:**
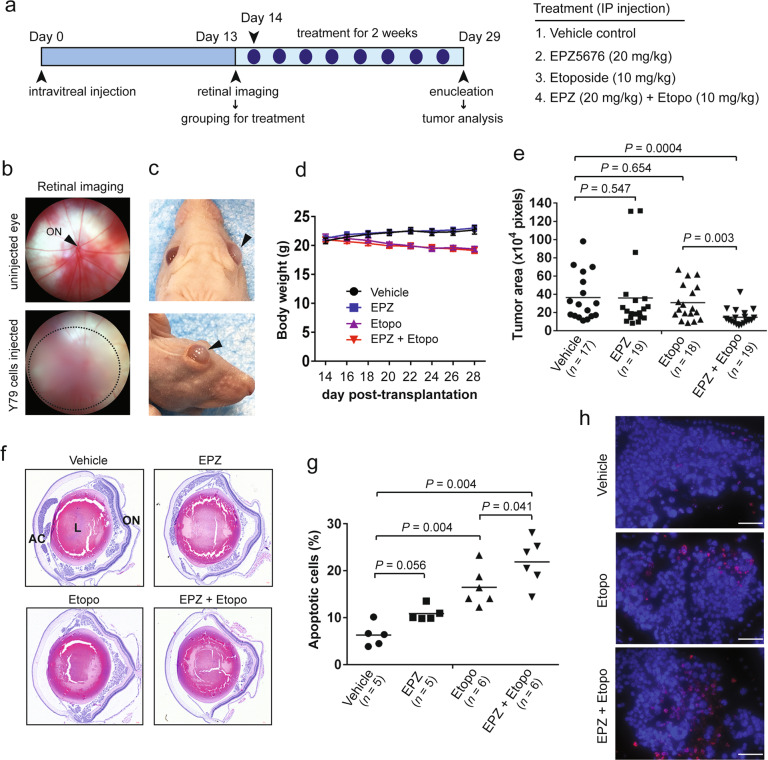
Cotreatment with a DOT1L inhibitor enhances the therapeutic efficacy of etoposide in orthotopic xenografts of RB. **a** Schematic of orthotopic xenograft study. **b** Retinal imaging to monitor tumor development on day 13 post-transplantation of Y79 cells. Xenografted eyes develop cloud-like tumors circled by dotted lines whereas uninjected eyes show a clear retinal view. ON optic nerve. **c** RB development in xenografted eyes indicated by an arrowhead. **d** Body weight changes of xenografted mice shown as the mean ± SEM. **e** Plot of average tumor areas in the indicated treatment groups. Data points on the plot represent the average tumor area per section per mouse (*n* = 17–19 mice per group), with the mean shown as the horizontal bar. The statistical analysis was performed by Mann–Whitney test (two-tailed). **f** Representative HE-stained images of tumor-burdened eyes from each treatment group. AC anterior chamber, L lens, ON optic nerve. **g** Quantification of apoptotic cells in xenografts after four rounds of the treatment. The percentage of apoptotic cells per eye was calculated by taking the average of TUNEL-positive nuclei/total nuclei from counting three random fields per eye. The statistical analysis was performed by Mann–Whitney test (two-tailed). **h** Representative TUNEL images for the indicated treatment groups. Scale bar: 50 µm.

Previous pharmacokinetic studies indicated that EPZ5676 has high intraperitoneal (IP) bioavailability in mice but also has high clearance in the plasma of mice and rats [33, 34]. In rat xenografts of MLL-rearranged leukemia, continuous intravenous (IV) infusion of EPZ5676 as a single agent resulted in dose-dependent tumor regression with a concomitant reduction in H3K79 methylation and consequential transcriptional repression of MLL target genes in the tumors [33]. A quantitative whole-body autoradiography study with radiolabeled EPZ5676 demonstrated that radioactivity can be detected in the eyes of rats at a relatively low level but nearly ten times higher than the lower limit of quantitation [34]. As continuous IV infusion of EPZ5676 is not practical as a therapy, we attempted a combination therapy with EPZ5676 and etoposide by IP administration on an orthotopic xenograft model of RB (Fig. 6a), under the hypothesis that EPZ5676-mediated chemosensitization to etoposide may not require a high dose of EPZ5676 delivered into the tumor-bearing eyes. The xenografted mice were examined for tumor establishment by retinal imaging before treatments (Fig. 6b), and untreated xenografts developed conspicuous tumors over 5–6 weeks after transplantation (Fig. 6c). During the treatment, some of the etoposide-treated mice experienced minor body weight loss (Fig. 6d). When tumor-burdened eyes were analyzed for an average tumor area to determine the therapeutic efficacy, single EPZ5676 or etoposide treatment was not efficacious for tumor regression whereas combined treatment significantly reduced the tumor areas and resulted in lesser variation in the final tumor size (Fig. 6e, f). The tumor size reduction in the cotreatment group did not involve HMGA2 downregulation in the tumors (Fig. S9), as epigenetic repression of DOT1L target genes would require continuous exposure to EPZ5676 as previously demonstrated [33]. Instead, we suspected that tumors subjected to the cotreatment might have undergone more robust apoptosis by the low dose of EPZ5676 delivered to the eyes with etoposide. To test this possibility, we performed TUNEL assays to determine the fraction of apoptotic cells over total cells (Fig. 6g, h). When we analyzed the final tumors after the complete treatment, many of the apoptotic tumor cells in the cotreatment group were found to be eliminated to the extent that the TUNEL signal was not clearly detectable (Fig. S10). To assess the ongoing apoptosis more accurately, we performed the assay on the xenografts after four rounds of the treatment (Fig. 6g, h). Both single etoposide and cotreatment groups displayed significantly higher apoptotic cells than vehicle-treated tumors (Fig. 6g), demonstrating that etoposide is the main apoptosis inducer in the tumors. Besides, the cotreatment group showed higher apoptotic cells than single etoposide group (Fig. 6g, h). Altogether, these data demonstrate that cotreatment with a DOT1L inhibitor enhances the efficacy of etoposide in orthotopic RB xenografts by making the response to etoposide more potent and consistent.

## Discussion

Epigenetic dysregulation contributes to RB tumorigenesis and malignant progression [1, 2]. Consistent with this notion, RB tumors possess aberrantly expressed chromatin regulators in contrast to normal retina, but their functions in RB development and possibilities as potential therapeutic targets have not been explored completely [4]. In this study, we investigated the role of histone H3K79 methyltransferase DOT1L in chemosensitization of RB cells. DOT1L was expressed in the majority of human RB in our cohorts whereas there was no detectable expression of DOT1L in normal retina, which would confer a selectivity for DOT1L targeting. However, single-agent therapy with a DOT1L inhibitor EPZ5676 was largely inefficacious for both RB cells and animal model, and presented several limitations including the requirement of high doses and sustainable drug bioavailability for significant anticancer effects. As DOT1L protein and its catalytic activity are known to be required for proper DNA damage response and repair [12, 18, 19, 27], combined treatments of EPZ5676 with other genotoxic agents were expected to potentiate the anticancer effects of the DNA-damaging agents. Indeed, DOT1L inhibition or downmodulation by gene knockdown sensitized RB cells to etoposide by enhancing apoptosis. In addition to verifying the role of DOT1L in DNA damage response and chemosensitization in RB cells, we discovered that HMGA2 is a novel DOT1L target gene in RB cells and its expression is epigenetically upregulated by DOT1L. Since HMGA2 has been known to promote RB cell proliferation and participate in the regulation of DNA damage response in cancer cells [21, 23, 24, 28], our findings altogether suggest that DOT1L inhibition has a dual role in chemosensitization of RB cells by directly impairing the early DNA damage response mediated by DOT1L itself upon genotoxic insults, and also by downregulating the expression of HMGA2 as a rather late effect of DOT1L inhibition.

DOT1L was identified to promote MLL-rearranged leukemia by interacting with various MLL-fusion proteins and thereby aberrantly upregulating the expression of MLL target genes [14, 15]. Although EPZ5676 monotherapy was efficacious for tumor regression in rat MLL-fusion xenografts, the therapeutic efficacy of EPZ5676 depended on the epigenetic repression of MLL target genes, which required continuous IV infusion to maintain the drug concentration in the plasma above a certain threshold [33]. In this study, we demonstrated that intermittent IP administration of EPZ5676 along with etoposide as a combination therapy significantly improves the therapeutic efficacy of etoposide in murine orthotopic xenografts of RB. These results suggested that the small dose of EPZ5676 delivered to the eyes might be sufficient to impair the function of DOT1L in early DNA damage response at DSB sites, consequentially augmenting the etoposide-induced apoptosis in the xenografts. Of note, the therapeutic efficacy of single etoposide treatment in the xenografts was highly variable, resulting in a wide range of the final tumor size. However, cotreatment with EPZ5676 has rendered the response to etoposide more stable and consistent, leading to lesser variation in the final tumor size. As predicted, the intermittent systemic administration of EPZ5676 was not sufficient to induce the epigenetic repression of HMGA2 in our xenografts. Since RB is a tumor for which local therapies such as intra-arterial and intravitreal chemotherapy can be safely performed in clinical settings [35, 36], localized administration of EPZ5676 may alleviate the limitations observed for the systemic IV administration, including the rapid clearance and inefficient drug delivery into the eyes. This approach may also allow for the epigenetic repression of HMGA2, thereby eliciting the dual effects of DOT1L inhibition in chemosensitization upon combination therapies with other drugs. Furthermore, locally delivered EPZ5676 is expected to target RB cells selectively without disrupting the retinal structure since normal retina does not express DOT1L and EPZ5676 by itself shows no signs of toxicity in our animal model. Therefore, it would be imperative to test local delivery routes into the eyes and examine the corresponding therapeutic efficacy of EPZ5676 in animal models of RB as a future direction.

In RB, targeted therapies are not available yet as a standard treatment option in clinics, and most chemotherapy regimens include conventional genotoxic or cytotoxic drugs. Despite the high efficacy of these conventional drugs in saving eyes and lives upon early diagnosis and timely treatment, RB occurs to young children who are still under the developmental process, and the heavy use of these genotoxic or cytotoxic drugs may pose a risk of developing multiple late effects later in their life. In this regard, targeting epigenetic regulators such as DOT1L may be beneficial to improve the efficacy of current chemotherapy by being able to reduce the doses of genotoxic drugs that are required to elicit the same response to therapy. In fact, a recent study revealed that dose reduction index (DRI), which compares the dose of single drug required to achieve the same effects with the dose used in combination therapy was greatly increased when topotecan was combined with an inhibitor of RAD51 polymerization in orthotopic RB xenografts [37]. Therefore, this approach is highly relevant to improve the current chemotherapy.

In summary, this study demonstrates that DOT1L targeting can sensitize RB cells to genotoxic drugs by impairing the dual functions of DOT1L manifested during early DNA damage response and epigenetic upregulation of HMGA2. Moreover, we presented the first preclinical evidence supporting that DOT1L targeting may be beneficial for combination therapies with standard chemotherapeutic drugs to improve the efficacy of current chemotherapy for RB.

## Materials and methods

### Cell culture and reagents

Y79 and Weri-Rb1 cells were obtained from American Type Culture Collection (ATCC, Manassas, VA, USA), and maintained in RPMI-1640 containing 10% FBS and penicillin–streptomycin (Invitrogen, Carlsbad, CA, USA) with routine testing for mycoplasma. EPZ5676, MS-275, and ciclopirox (CPX) were purchased from Selleck (Houston, TX, USA). These drugs and etoposide (Sigma, St. Louis, MO, USA) were dissolved in dimethyl sulfoxide (DMSO). When cells were treated with drugs for a longer time up to 8 days, drugs were replenished every two days under nonsaturating culture conditions. Adenoviruses for HMGA2 expression (VH893703) were purchased from Viagene Biosciences (Rockville, MD, USA). Lentiviral knockdown for DOT1L and HMGA2 was performed using the TRC lentiviral shRNA clones from Horizon Discovery (St. Louis, MO, USA) and the mature antisense sequences are as follows:

shDOT1L #210: TTGTTTAGCTTCTTCTTGCGG

shDOT1L #211: ATAGCGAGCTTGAGATCCGGG

shDOT1L #213: TAGCTCCACAATGCTGATCTG

shHMGA2 #965: TTCTGAACAACTTGTTGTGGC

shHMGA2 #966: TTGAGCTGCTTTAGAGGGACT

### Lentiviral gene knockdown

The lentiviral particles were produced by following the pLKO.1 packaging protocol from Addgene. Lentiviral transduction was performed for 12 h after plating 6 × 10^6^ suspension RB cells on poly-d-lysine (0.1 mg/ml, Sigma)-coated dishes. After the 12 h of infection, cells were incubated for 3–4 days in regular culture media and then used for experiments immediately.

### Human retinoblastoma tissues

Human RB tissues were obtained from the ocular tumor division and department of pathology at the Zhongshan Ophthalmic Center (ZOC). The study with human clinical samples conformed to the standards set by the Declaration of Helsinki, and was approved by the ZOC institutional review board. All human specimens used for this study were de-identified, and written informed consent forms were obtained.

### Immunohistochemistry

Serial sections of human RB were subjected to immunostaining with anti-DOT1L (A300-953A; Bethyl, Montgomery, TX, USA) and anti-HMGA2 (8179; Cell Signaling Technology, Danvers, MA, USA) antibodies, followed by nuclear counterstaining with hematoxylin.

### Cell viability assay

Cell viability and proliferation in response to drug treatments were determined by MTT assays (Sigma). Cells were plated at a density of 8 × 10^3^ cells/96-well in triplicate, and the IC_50_ values of drugs were calculated by fitting the dose response curve from at least five independent assays with GraphPad Prism (GraphPad Software, La Jolla, CA, USA).

### Western blot

Cleared lysates (20–25 µg) were subjected to 7.5 or 12.5% SDS-PAGE. Antibodies for western blots are as follows: DOT1L (77087), HMGA2 (8179), γH2AX (9718), cleaved PARP (9541), phospho-ATM (Ser1981) (5883), ATM (2873), phospho-CHK2 (Thr68) (2197), CHK2 (2662), phospho-ATR (Ser428) (2853), ATR (2790), phospho-CHK1 (Ser296) (90178), CHK1 (2360), total histone H3 (9715) from Cell Signaling Technology; dimethyl-histone H3 (Lys79) (ab3594; Abcam, Cambridge, MA, USA), actin (A1978; Sigma), and caspase-3 (40924; Active Motif, Carlsbad, CA, USA).

### Chromatin immunoprecipitation (ChIP) and quantitative RT-PCR (qRT-PCR)

ChIP was performed by using SimpleChIP Enzymatic Chromatin IP kit (Cell Signaling Technology) according to the manufacturer’s instruction. The antibodies used for ChIP are as follows: DOT1L (77087) and total histone H3 (4620) from Cell Signaling Technology; dimethyl-histone H3 (Lys79) (ab3594; Abcam), and RNA polymerase II (05-623; Millipore, Burlington, MA, USA). ChIP DNA was analyzed by qPCR along with the 5% input chromatin that was saved and processed together with the ChIP samples, and quantified as % input for each target before calculation of fold changes or ratios of the signals. The qRT-PCR was performed with at least three independent sets of cDNA. The primer sequences for the qRT-PCR and ChIP assays are listed in supplementary information (Table S2).

### RNA-sequencing analysis

Using total RNA from Y79 cells treated with 10 µM EPZ5676 for 6 days, RNA-seq libraries were prepared by the standard Illumina protocols and subjected to 150 bp paired-end sequencing on an Illumina HiSeq 2500 platform by BerryGenomics (Beijing, China). The differentially expressed genes (DEGs) between vehicle (DMSO) and EPZ5676-treated cells were detected by edgeR package, based on the analysis criteria of ≥2-fold change in expression and a false discovery rate of 0.05. The KEGG pathway enrichment analysis of DEG sets was implemented by the KOBAS 3.0 package using the database downloaded from kegg.jp, and the pathways with an adjusted *p*-value below 0.05 were considered as significantly enriched unless indicated otherwise. The Gene Ontology (GO) analysis of DEGs was performed by topGO package using the GO database downloaded from geneontology.org with a *p*-value <0.05. The DEGs belonging to a relevant enriched pathway or GO term of interest were further selected for a heat map analysis. The RNA-seq data in this study were deposited in the NCBI Gene Expression Omnibus (GEO) database under the accession number GSE174167.

### Terminal deoxynucleotidyl transferase-dUTP nick end labeling (TUNEL) assay

Paraffin-embedded tissue sections were deparaffinized, followed by permeabilization in proteinase K solution (20 µg/ml proteinase K in 10 mM Tris-Cl, pH 7.4) for 20 min at 37 °C before the TUNEL assay with Click-iT TUNEL Alexa Fluor Imaging Assay kit (Invitrogen) according to the manufacturer’s protocol. Three random fields with viable tumor cells in the xenograft sections were taken for the quantification of TUNEL-positive apoptotic cells per eye. The percentage of apoptotic cells per tumor-burdened eye was calculated by taking the average of TUNEL-positive nuclei/total nuclei in the field.

### Therapeutic study on orthotopic xenografts

Orthotopic xenografts of RB were established according to the procedure described previously [10]. Briefly, Y79 cells (2 × 10^5^ in 2 µl) were injected into the vitreous of the right eye while leaving the left eye uninjected as a control, using 7 week-old BALB/c female nude mice (Model Animal Research Center, Nanjing University). On day 13 post-intravitreal transplantation, tumor formation was examined by retinal imaging with a Micron IV retinal microscope (Phoenix Research Lab, Pleasanton, CA, USA). Only the mice with well-established tumors (covering >50% of the whole fundus by retinal imaging) were divided into four treatment groups with a similar tumor burden. Single and combined treatments with EPZ5676 (20 mg/kg) and etoposide (10 mg/kg) prepared in 2% DMSO/30% polyethylene glycol 300/PBS as a vehicle were performed by intraperitoneal (IP) injection every other day for two weeks. Tumor-burdened eyes were analyzed for the average tumor area per eye by following the procedure described previously [10], except for the mice with incomplete treatment due to unexpected death. Some of the xenograft sections were subjected to TUNEL assays or immunostaining with anti-HMGA2 antibody (8179; Cell Signaling Technology). All animal studies were conducted with the approval of the Sun Yat-sen University Institutional Animal Care and Use Committee.

### Statistical analysis

Statistical significance was determined from at least three independent experiments by unpaired Student’s *t*-test (two-tailed) using GraphPad Prism. For in vivo work, one-way ANOVA test was performed first to determine the statistical difference among the multiple groups, and the differences between two comparison groups were analyzed by Mann–Whitney test (two-tailed).

## Supplementary information

Supplementary information

Supplementary Table S1

## Data Availability

The RNA-seq data in this study were deposited in the NCBI Gene Expression Omnibus (GEO) database under the accession number GSE174167. All other datasets used during the current study are available from the corresponding author on reasonable request.
