# Supplementary material for: Targeting of histone methyltransferase DOT1L plays a dual role in chemosensitization of retinoblastoma cells and enhances the efficacy of chemotherapy
Source: Cell Death Dis. 2021 Dec 9;12(12):1141. doi: 10.1038/s41419-021-04431-y (PMC8660841; doi:10.1038/s41419-021-04431-y)
Supplement: Supplementary file 1 — Supplementary information [file 41419_2021_4431_MOESM1_ESM.pdf]

Supplementary Information

Supplementary Table S1. List of differentially expressed genes in EPZ5676-treated Y79 cells relative to the vehicle-treated control.

Due to the space limit, Supplementary Table S1 is provided as a separate file.

Supplementary Table S2. Primers used in this study

**Quantitative RT-PCR**	
(1) DOT1L	Product size
Forward: 5'-CCACCAACTGCAAACATCAC-3'	
Reverse: 5'-AGAGGAAATCGCCTCTCTCC-3'	147 bp
(2) HMGA2	
Forward: 5'-ACCCAGGGGAAGACCCAAA-3'	
Reverse: 5'-CCTCTTGGCCGTTTTTCTCCA-3'	93 bp
(3) FOXO1	
Forward: 5'-TCGTCATAATCTGTCCCTACACA-3'	
Reverse: 5'-CGGCTTCGGCTCTTAGCAAA-3'	168 bp
(4) SIX1	
Forward: 5'-CTGCCGTCGTTTGGCTTTAC-3'	
Reverse: 5'-GCTCTCGTTCTTGTGCAGGT-3'	135 bp
(5) HOXA11	
Forward: 5'-TGCCAAGTTGTACTTACTACGTC-3'	
Reverse: 5'-GTTGGAGGAGTAGGAGTATGTCA-3'	107 bp
(6) Actin	
Forward: 5'-AGAGCTACGAGCTGCCTGAC-3'	
Reverse: 5'-AGCACTGTGTTGGCGTAC-3'	184 bp
(7) NGFR	
Forward: 5'-CCTACGGCTACTACCAGGATG-3'	
Reverse: 5'-CACACGGTGTTCTGCTTGT-3'	109 bp
(8) FLT1	
Forward: 5'-GAAAACGCATAATCTGGGACAGT-3'	
Reverse: 5'-GCGTGGTGTGCTTATTTGGA-3'	173 bp
(9) MEIS1	
Forward: 5'-GATATAGCCGTGTTCGCCAAA-3'	
Reverse: 5'-CGGTGGCAGAAATTGTCACAT-3'	158 bp
(10) ESRRG	
Forward: 5'-GCCCTCACTACACTGTGTGAC-3'	
Reverse: 5'-CCTGCTAATTTGGACTGGTCTT-3'	233 bp
(11) PROX1	
Forward: 5'-AAAGGACGGTAGGGACAGCAT-3'	
Reverse: 5'-CCTTGGGGATTCATGGCACTAA-3'	76 bp
(12) LHX3	
Forward: 5'-CAGTATTTCCGCAACATGAAGC-3'	
Reverse: 5'-GCTCCCGTAGAGGCCATTG-3'	150 bp

**ChIP-PCR**	
(1) HMGA2-A	
Forward: 5'-GCCGAGGCAGTTGTATTTCG-3'	
Reverse: 5'-AGTATGAGGAGGAGGGAGGC-3'	90 bp
(2) HMGA2-B	
Forward: 5'-TCATCCCACTTGAATCTTGGGG-3'	
Reverse: 5'-AGCTCCTGAGTCTTGCACCA-3'	81 bp
(3) HMGA2-C	
Forward: 5'-CCCGCCTAACATTTCAAGGG-3'	
Reverse: 5'-GTTGGGATCAGGAGTTGCGG-3'	98 bp
(4) CDKN2A	
Forward: 5'-GGGCTCTCACAACTAGGAAAG-3'	
Reverse:5'-GGGTGTTTGGTGTCATAGGG-3'	86 bp

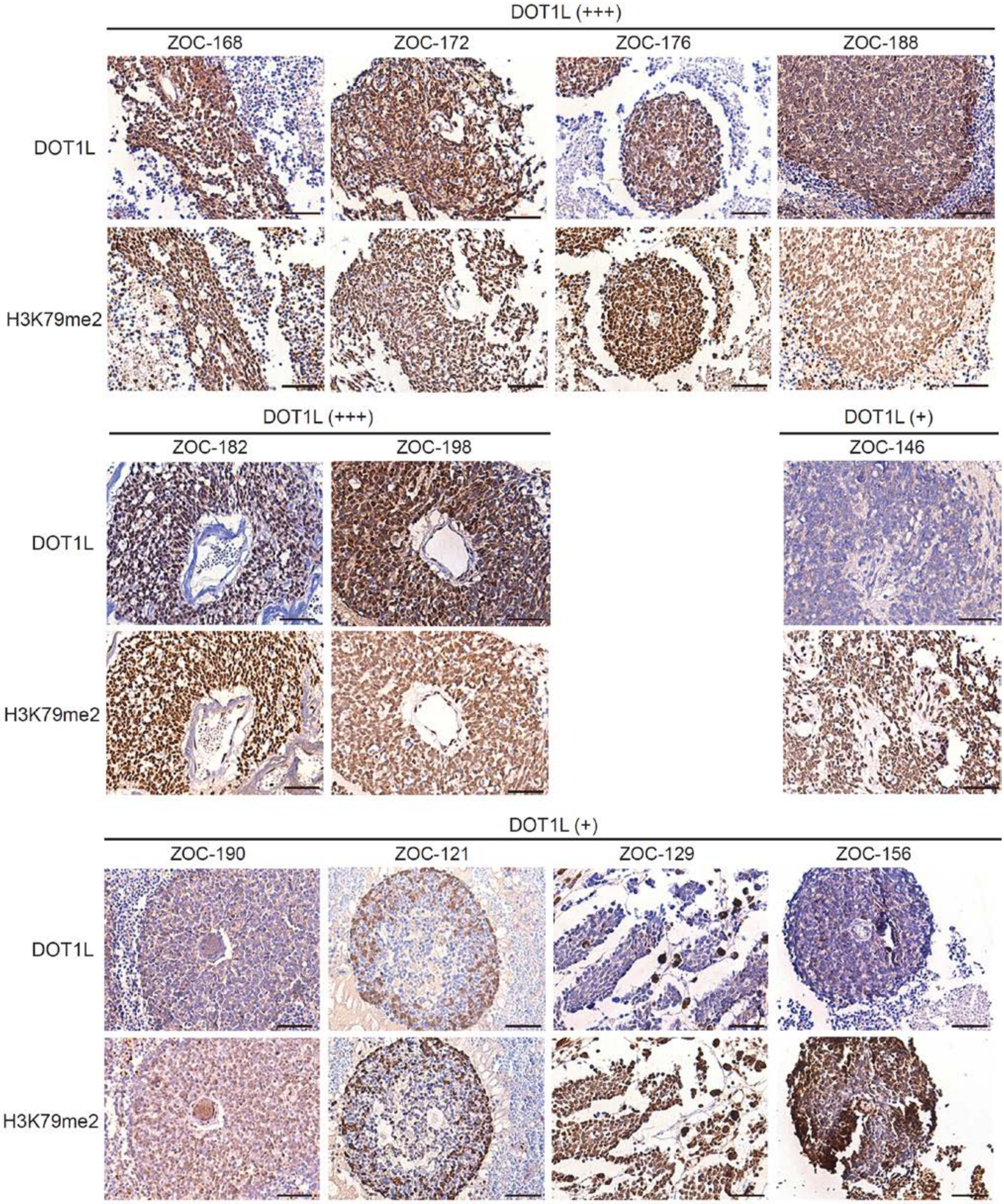

Supplementary Fig. S1. (continued)

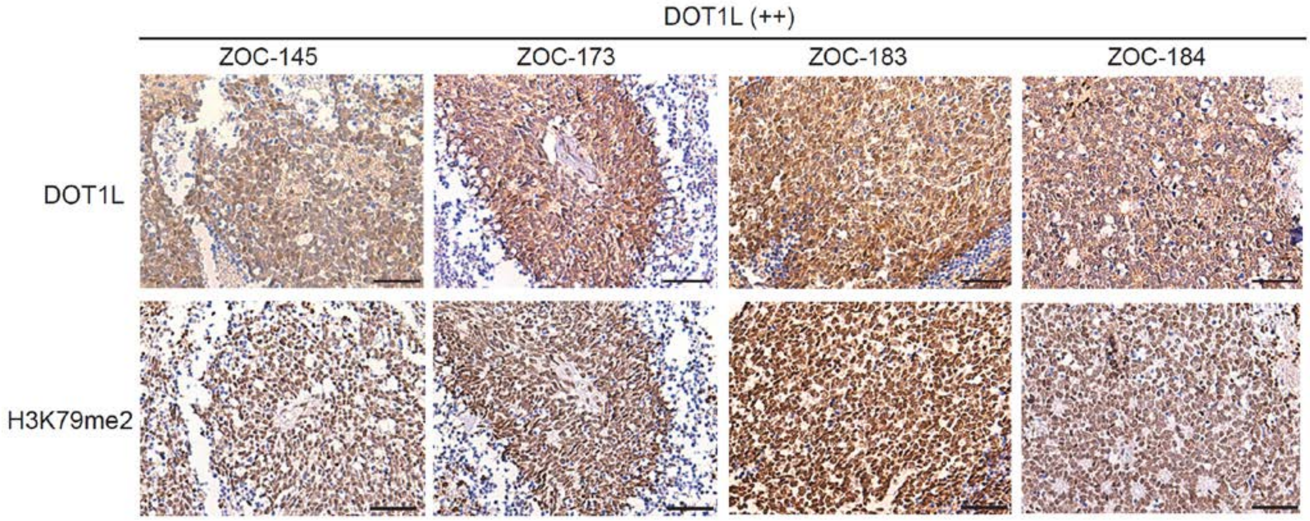

Supplementary Fig. S1. Expression of DOT1L and H3K79me2 in human RB. Serial sections of the indicated tumor tissues were immunostained for DOT1L and H3K79me2. Nuclei were counterstained with hematoxylin. Scale bar: 50 μm.

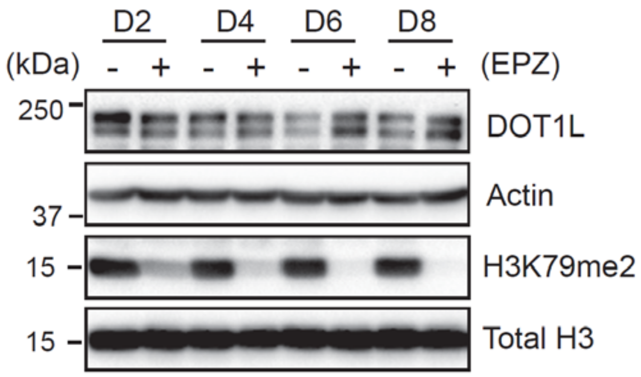

Supplementary Fig. S2. DOT1L protein levels in EPZ5676-treated Y79 cells at indicated time points. Cells were treated with 10 µM EPZ5676 continuously up to 8 days. The drug was freshly added every two days with a medium change, and the cells were maintained under nonsaturating culture conditions during the whole treatment time.

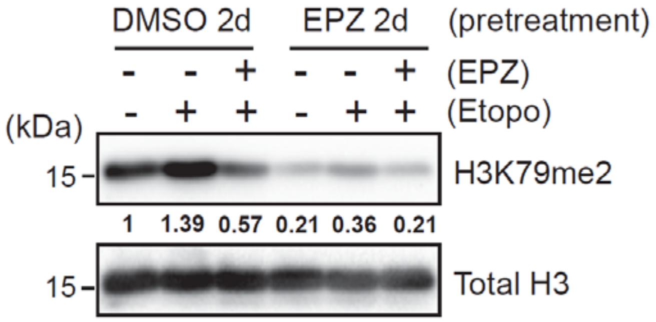

Supplementary Fig. S3. H3K79me2 is increased by etoposide treatment in both DMSO and EPZ5676-pretreated cells. Y79 cells were pretreated with either DMSO or 10 μM EPZ5676 for 2 days. After EPZ5676 was washed away, the pretreated cells were subjected to a single or combined treatment of 0.2 μM etoposide and 10 μM EPZ5676 for 48 h. Densitometric analysis results for the H3K79me2/total H3 are shown with the immunoblot.

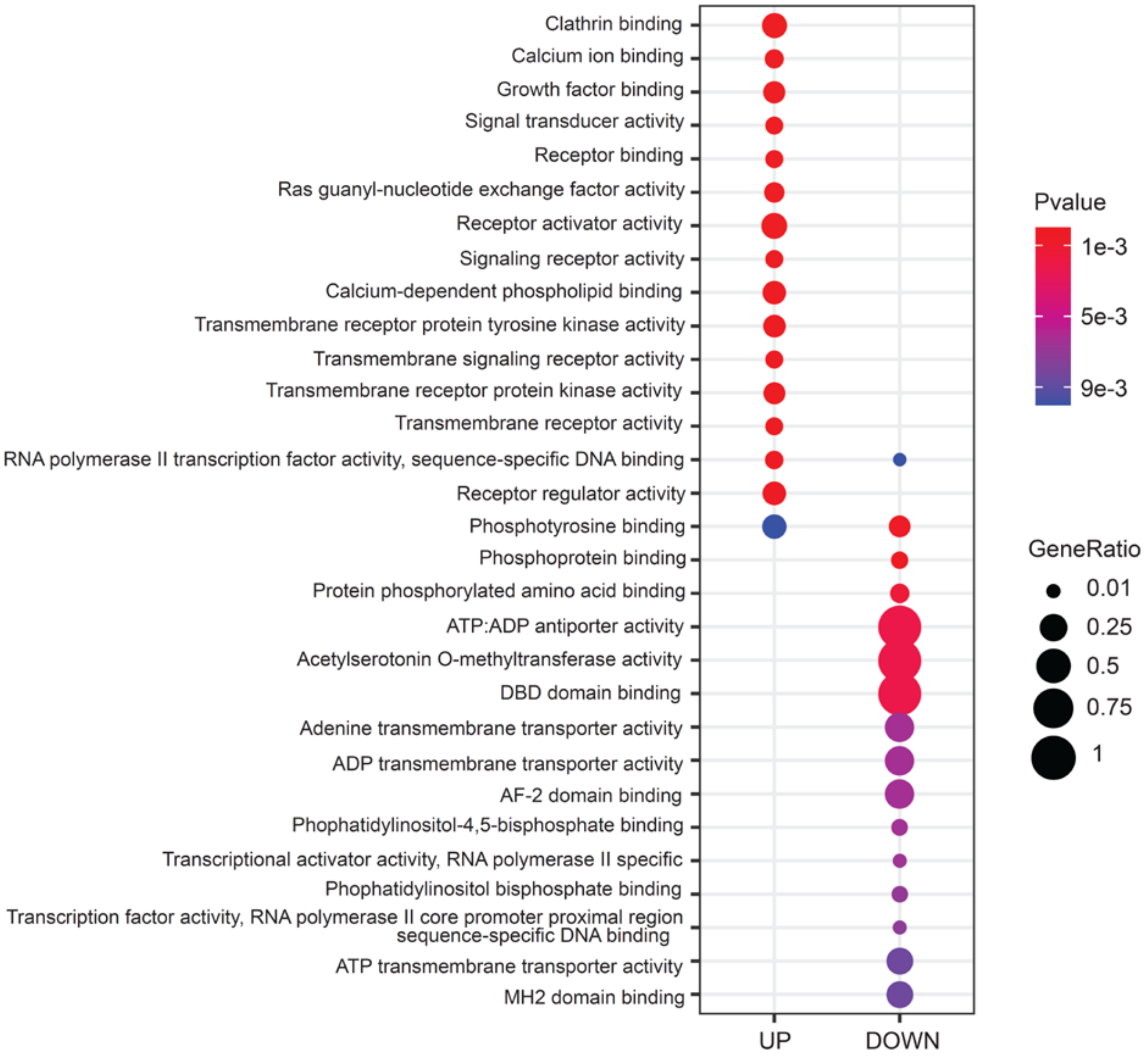

Supplementary Fig. S4. Gene ontology (GO) analysis of differentially expressed genes (DEGs) in EPZ5676-treated Y79 cells. The top 15 enriched GO terms of molecular function are ranked by p-values, and shown for upregulated (UP) and downregulated (DOWN) genes as a split dot plot. The GO terms which are common for both UP and DOWN genes are displayed as double dots. The size of dots on the plot indicates the gene count ratio of DEG/annotated genes belonging to each GO term listed.

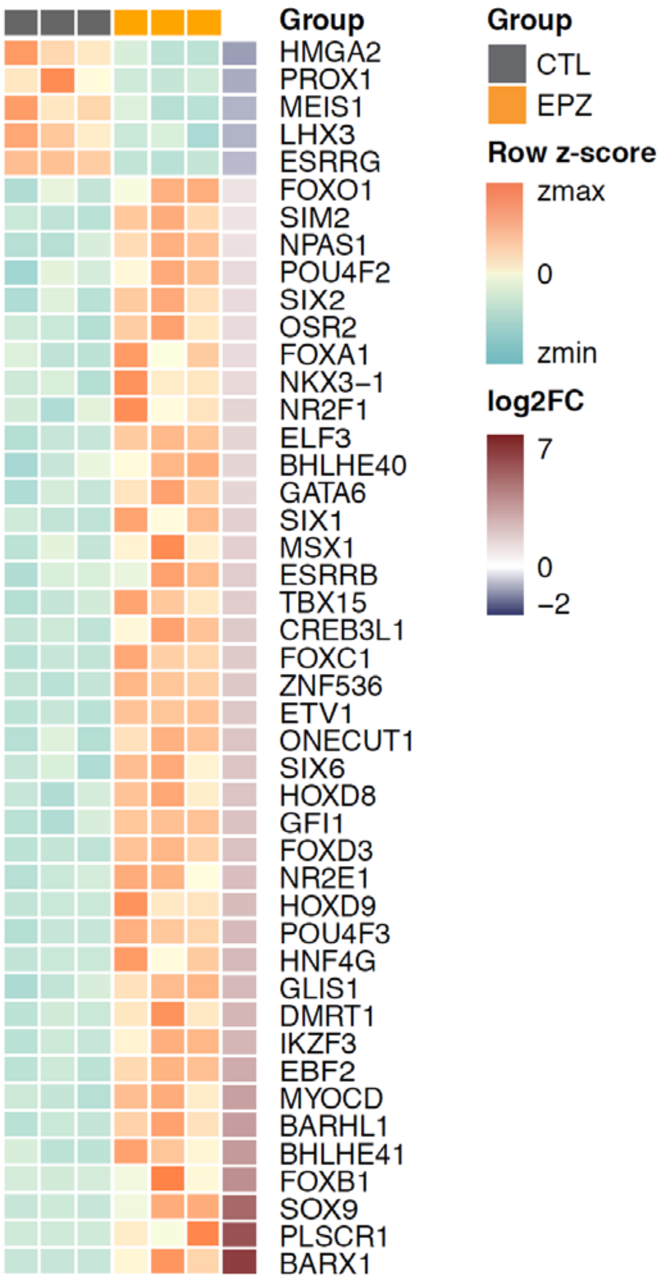

Supplementary Fig. S5. Heat map of DEGs belonging to the GO term of RNA polymerase II transcription factor activity/sequence-specific DNA binding. Each column represents an independent replicate.

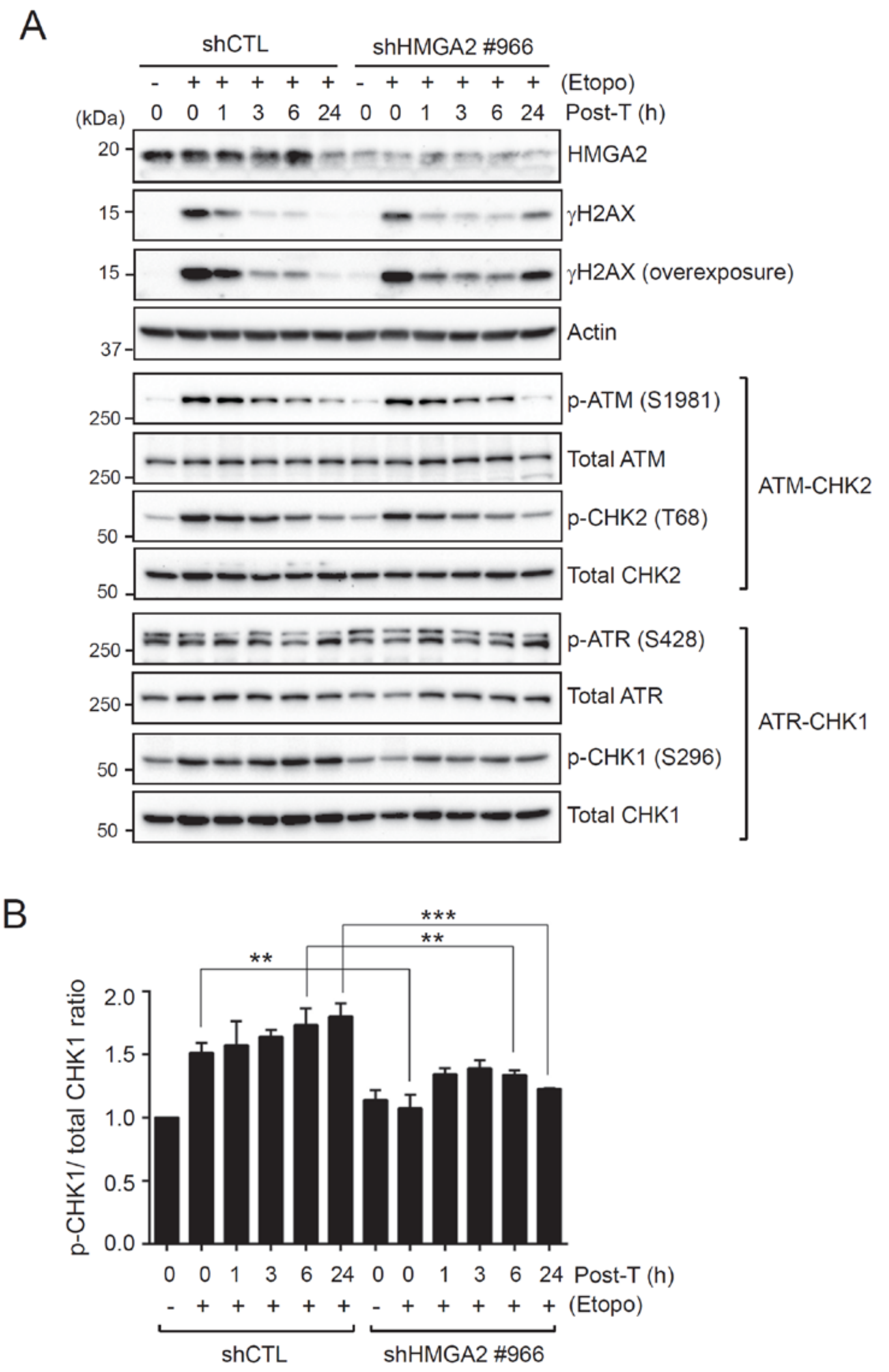

Supplementary Fig. S6. CHK1 phosphorylation is reduced in HMGA2-knockdown Y79 cells during the DNA damage response induced by etoposide treatment. (A) Immunoblots for indicated proteins showing the recovery kinetics from the acute DNA damage induced by etoposide. Cells were treated with 10 μM etoposide for 1 h, and then placed in fresh media without the drug for the indicated time post-treatment (post-T) to allow the recovery. (B) Densitometric analysis of p-CHK1/total CHK1 levels in (A). The graph represents the mean ± SD (N = 3). ** $P < 0.01$, *** $P < 0.001$: unpaired Student's *t*-test (two-tailed).

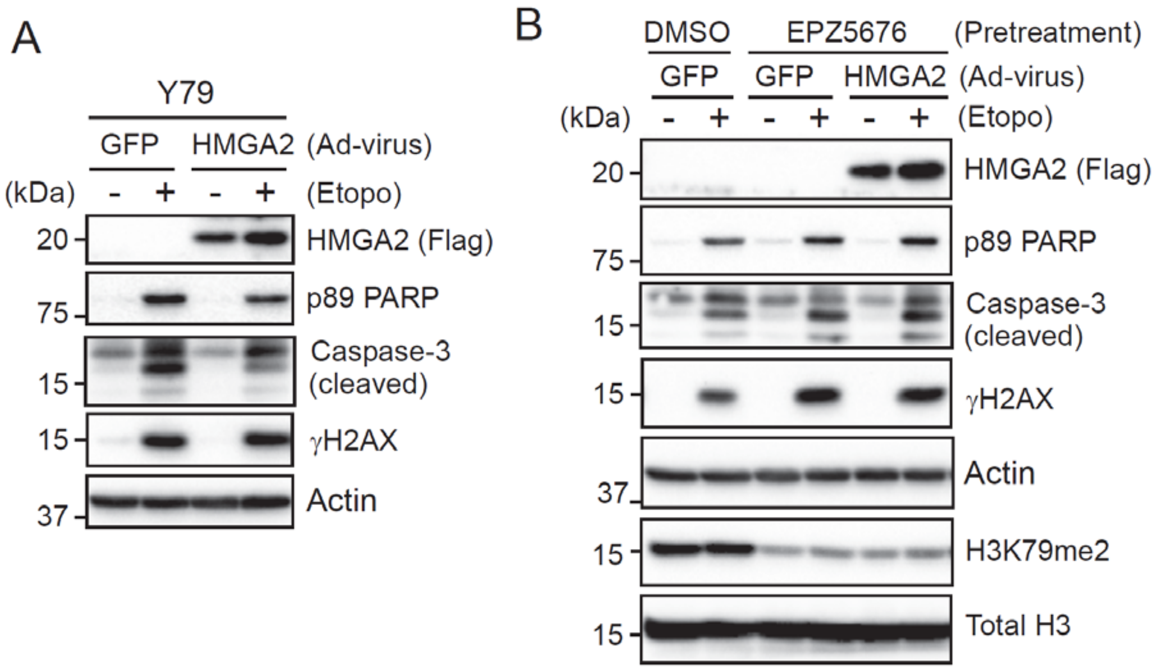

Supplementary Fig. S7. HMGA2 overexpression in EPZ5676-treated cells does not attenuate the etoposide-induced apoptosis. (A) Decreased apoptosis in response to etoposide upon HMGA2 overexpression. At 24 h post-adenoviral HMGA2 or GFP transduction in Y79 cells, treatment with either vehicle or 10 μM etoposide was performed for 24 h. (B) HMGA2 overexpression in EPZ5676-pretreated cells does not attenuate the etoposide-induced apoptosis. Y79 cells were pretreated with either DMSO or 10 μM EPZ5676 for 2 days, and then subjected to the indicated adenoviral transduction. After 24 h, the cells were treated with 5 μM etoposide for 24 h.

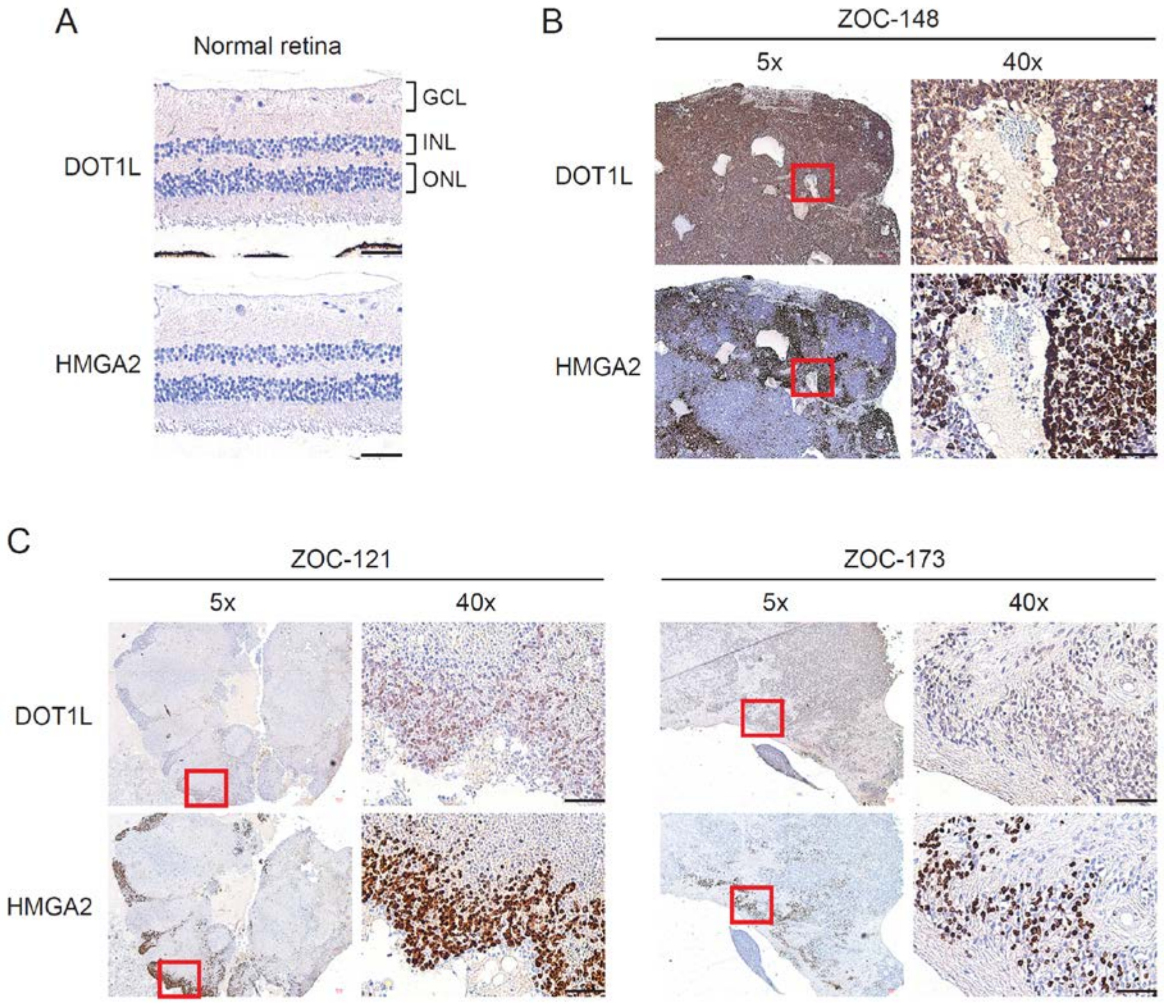

Supplementary Fig. S8. Expression of DOT1L and HMGA2 in human RB. (A-C) Immunostaining of DOT1L and HMGA2 in human RB and normal retina (42 years of age). Two serial sections of indicated tumor tissues were immunostained for DOT1L and HMGA2, respectively. The same region of the serial sections (marked by a red square) was magnified to visualize the nuclear staining of DOT1L and HMGA2 in the tumor on the right (40x). Nuclei were counterstained with hematoxylin. GCL: ganglion cell layer; INL: inner nuclear layer; ONL: outer nuclear layer; Scale bar: 50 μm.

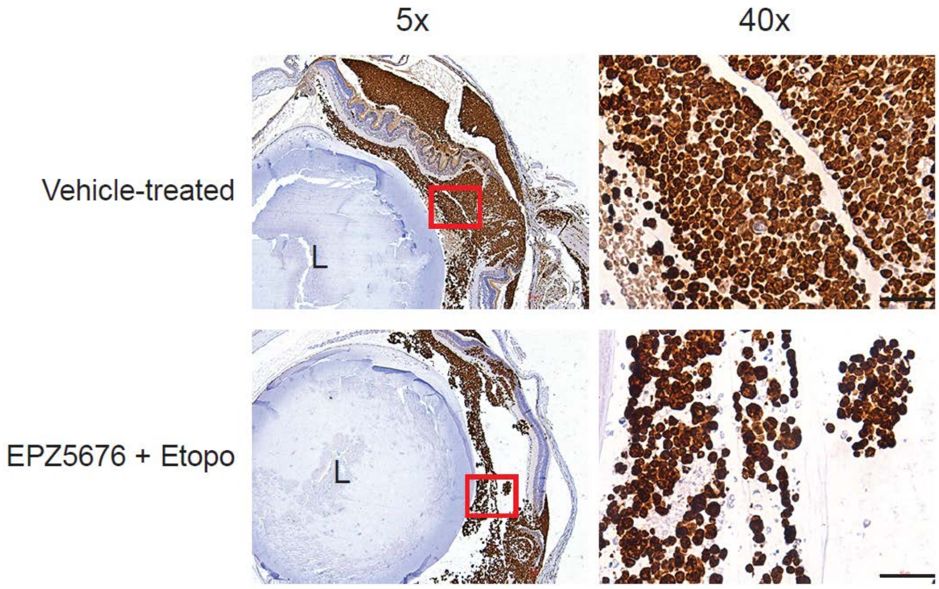

Supplementary Fig. S9. Expression of HMGA2 in xenografted tumors after treatments. Xenografted tumor sections after completion of the indicated treatments for two weeks were immunostained for HMGA2. A region of the sections (marked by a red square) was magnified to visualize the nuclear staining of HMGA2 on the right (40x). Nuclei were counterstained with hematoxylin. L: lens; Scale bar: 50 μm.

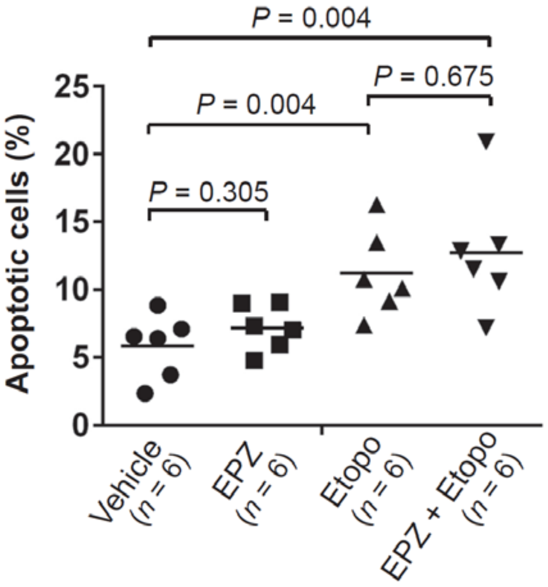

Supplementary Fig. S10. Quantification of apoptotic cells in the final xenografted tumors after complete treatments. The percentage of apoptotic cells per eye was calculated by taking the average of TUNEL-positive nuclei/total nuclei from counting three random fields per eye. The statistical analysis was performed by Mann-Whitney test (two-tailed).
